# SCRaMbLE generates designed combinatorial stochastic diversity in synthetic chromosomes

**DOI:** 10.1101/gr.193433.115

**Published:** 2016-01

**Authors:** Yue Shen, Giovanni Stracquadanio, Yun Wang, Kun Yang, Leslie A. Mitchell, Yaxin Xue, Yizhi Cai, Tai Chen, Jessica S. Dymond, Kang Kang, Jianhui Gong, Xiaofan Zeng, Yongfen Zhang, Yingrui Li, Qiang Feng, Xun Xu, Jun Wang, Jian Wang, Huanming Yang, Jef D. Boeke, Joel S. Bader

**Affiliations:** 1 BGI-Shenzhen, Shenzhen 518083, China;; 2 Centre for Synthetic and Systems Biology, School of Biological Sciences, University of Edinburgh, Edinburgh EH9 3JL, United Kingdom;; 3 High-Throughput Biology Center, School of Medicine, Johns Hopkins University, Baltimore, Maryland 21205, USA;; 4 Department of Biomedical Engineering, School of Engineering, Johns Hopkins University, Baltimore, Maryland 21218, USA;; 5 Department of Biochemistry and Molecular Pharmacology and Institute for Systems Genetics, NYU Langone Medical Center, New York, New York 10016, USA;; 6 Department of Biology, University of Copenhagen, DK-2200 Copenhagen, Denmark;; 7 Princess Al Jawhara Center of Excellence in the Research of Hereditary Disorders, King Abdulaziz University, Jeddah 21589, Saudi Arabia;; 8 James D. Watson Institute of Genome Science, Hangzhou 310058, China

## Abstract

Synthetic chromosome rearrangement and modification by *loxP*-mediated evolution (SCRaMbLE) generates combinatorial genomic diversity through rearrangements at designed recombinase sites. We applied SCRaMbLE to yeast synthetic chromosome arm *synIXR* (43 recombinase sites) and then used a computational pipeline to infer or unscramble the sequence of recombinations that created the observed genomes. Deep sequencing of 64 *synIXR* SCRaMbLE strains revealed 156 deletions, 89 inversions, 94 duplications, and 55 additional complex rearrangements; several duplications are consistent with a double rolling circle mechanism. Every SCRaMbLE strain was unique, validating the capability of SCRaMbLE to explore a diverse space of genomes. Rearrangements occurred exclusively at designed *loxPsym* sites, with no significant evidence for ectopic rearrangements or mutations involving synthetic regions, the 99% nonsynthetic nuclear genome, or the mitochondrial genome. Deletion frequencies identified genes required for viability or fast growth. Replacement of 3′ UTR by non-UTR sequence had surprisingly little effect on fitness. SCRaMbLE generates genome diversity in designated regions, reveals fitness constraints, and should scale to simultaneous evolution of multiple synthetic chromosomes.

Rapid gains in DNA synthesis technologies have created new capabilities for understanding the structure, function, and evolution of genomes. Pioneering work established that native genomic DNA can be functionally replaced by synthetic DNA molecules encoding identical sequences. Milestones include the 7.5-kb synthetic poliovirus in 2002 (Cello et al. 2002), the 5.4-kb synthetic φX174 phage in 2003 (Smith et al. 2003), and the 1.1 Mbp “synthia” *Mycoplasma genitalium* genome in 2008 (Gibson et al. 2008). Synthetic genomes offer the possibility of redesign to improve their value for research and engineering applications, notably the full synthesis of the 40-kb refactored T7 phage in 2005 (Chan et al. 2005) and the genome-scale editing of *Escherichia coli* MG1655 to recode all UAG stop codons to UAA (Lajoie et al. 2013).

Eukaryotic synthetic genomics has centered on *Saccharomyces cerevisiae* (yeast), simultaneously a powerful model organism and a producer of valuable products. Yeast genome synthesis provides access to genome biology relating to features such as chromatin structure, splicing, and linear chromosomes with telomeres, centromeres, and recombinations that are absent from prokaryotes. With the goal of answering these questions, the Sc2.0 Project has designed and synthesized synthetic chromosomes that function in yeast (Dymond et al. 2011; Annaluru et al. 2014)

Beyond recapitulating native biology with a synthetic DNA molecule that is an exact copy of a natural sequence, synthetic genomics afford the possibility of designer genome features that can be exploited to learn biology and introduce valuable new capabilities. Yeast chromosomes designed as part of the Sc2.0 Project include designed site-specific recombination targets, termed *loxPsym* sites, which are substrates for an inducible form of the appropriate site-specific recombinase, Cre-EBD (Lindstrom and Gottschling 2009). Unlike the native directional *loxP* site, which permits a single orientation for recombination, the synthetic *loxPsym* site's symmetry ensures that any pair of sites can recombine in either orientation (Hoess et al. 1986). Controlled expression of Cre-EBD may then lead to stochastic rearrangements of chromosome segments flanked by *loxPsym* sites, with deletions and inversions in principle equally likely based on the relative orientation of the *loxPsym* sites in the recombination junction.

Previous work demonstrated the ability of this system, synthetic chromosome rearrangement and modification by *loxPsym*-mediated evolution (SCRaMbLE), to generate phenotype diversity by application to *synIXR*, a circular synthetic chromosome based on the right arm of yeast Chromosome IX. Strains generated by SCRaMbLE had heterogeneous growth rates, and auxotrophic mutants corresponded to deletions of specific *loxPsym*-flanked regions containing known candidate genes. Deletions provide a route for a population of cells to explore condition-specific minimal genomes, a complementary approach to expert-inferred or designed minimal genomes (Koonin 2000; Suzuki et al. 2015). Inversions and more complicated rearrangements could provide selectable diversity for rapid directed evolution.

While initial applications of SCRaMbLE have been promising (Dymond and Boeke 2012), many potential challenges exist. First, introducing multiple *loxPsym* sites may create genome instability through homologous recombination even in the absence of Cre recombinase; subsequent to the end of Cre induction, leaky expression or continuing protein activity may lead to instability. When Cre is active, ectopic recombinations may involve “off-target” or cryptic sites in the yeast genome, outside the designed *loxPsym* sites; Cre generates ectopic recombinations between *loxP* sites and off-target sites, albeit at extremely low frequency (Sauer 1992). For desired recombinations at *loxPsym* sites, random pairing is desirable for maximum diversity; beyond the 82-bp minimum distance required for *loxP* recombination (Hoess et al. 1985), recombination hotpots may reduce the diversity. Detailed characterization of the genomes of SCRaMbLE strains are required to answer these questions, but even here the genome rearrangements generated by SCRaMbLE may not be amenable to standard genome sequencing and assembly methods.

Here we test these crucial hypotheses. As described below, we sequenced the genomes of 64 *synIXR* SCRaMbLE strains, including the nonsynthetic as well as synthetic chromosomes to detect ectopic recombinations; we also examined genome stability for different Cre induction systems. We characterized in detail the types of recombinations detected, including deletions, inversions, and a surprisingly high frequency of duplications. Our results verify the utility of SCRaMbLE to generate combinatorial diversity on demand.

## Results

### Chromosome design and nomenclature

**Figure 1.**
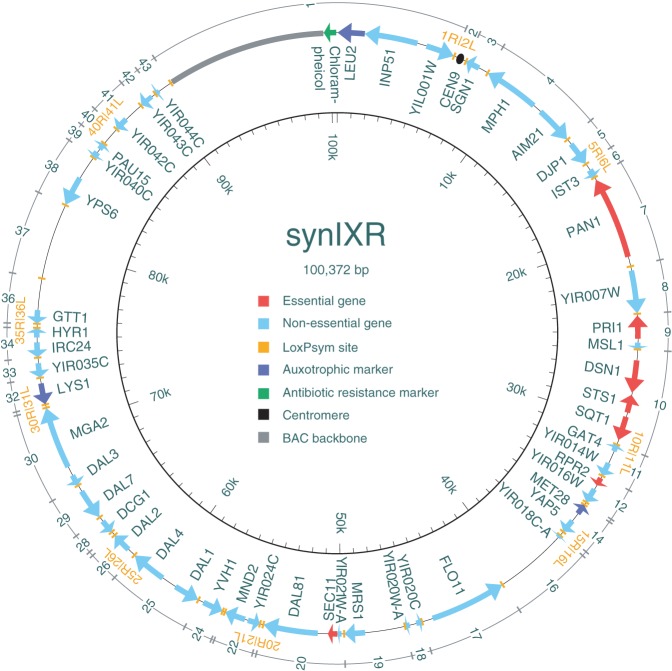
The circular *synIXR* synthetic chromosome comprises 43 segments, numbered consecutively as shown, with *loxPsym* sites (orange bars) serving as junctions between adjacent segments. Arrows indicate genes, with colors denoting essential genes (red), auxotrophic markers (purple), other nonessential genes (light blue), and the chloramphenicol resistance marker (green). The centromere, in segment 2, is shown as a black circle. The *left* and *right* ends of each segment are denoted “L” and “R,” and *loxPsym* sites are denoted by the unique half-sites of the segments they join. For example, the *loxPsym* site at the junction between segments 1 and 2 involves half-sites 1R and 2L.

SCRaMbLE is designed to generate diversity by combinatorial rearrangement of segments flanked by designed recombination sites. The original segments are represented as consecutive integers, one through 43 for *synIXR* (Fig. 1). The *loxPsym* junctions are denoted by the unique left (L) and right (R) ends of the segments they join. After the SCRaMbLE process, the rearranged chromosome is represented using standard gene order conventions as a list of the segments in their new order, with junctions connecting adjacent segments and an implicit additional junction between the final and initial segment for the circular *synIXR* chromosome. Deletions and duplications change the number of times that a segment appears, and inversions change the signs and reverse the order of the affected region.

### Chromosome SCRaMbLE and selection

A total of 63 *synIXR* SCRaMbLE colonies were previously generated with Cre introduced transiently on a plasmid and then selected to have auxotrophies arising from loss of function of *LYS1* or *MET28* encoded in the synthetic region, with 33 Lys^−^ (*LYS1/YIR034C*, segment 32), 20 Met^−^ (*MET28/YIR017C*, segment 14), and 10 Met^−^ Lys^−^ (Dymond et al. 2011). All genotypes were consistent with phenotypes, with one or both of *LYS1* and *MET28* deleted (Supplemental Table S1). The four semi-*synVIL* SCRaMbLE colonies were not subject to selection but were exposed to SCRaMbLE for a long period of time intentionally to characterize the maximum viable DNA deletion (Dymond and Boeke 2012).

The strain JS274 had the Cre-EBD expression cassette integrated at the *HO* locus. While this strain was not intentionally induced, it showed evidence for leaky expression of recombinase, consistent with earlier findings (Smith et al. 2003): While initially phenotyped as Lys^−^, it lost segment 14 (containing *MET28*) prior to sequencing. Counts of SCRaMbLE events and junctions include this strain.

### Genome sequencing

We sequenced DNA from one wild-type strain (BY4741), 64 *synIXR* SCRaMbLE strains, and two non-SCRaMbLE parental strains. For each strain, short-insert libraries (average insert size, 500 bp) were generated without PCR amplification to avoid creating artifacts through crossover PCR at *loxPsym* sites. Instead, yeast cultures were grown to saturation, and amplicon-free libraries were prepared. For 10 strains whose rearrangements were too complex to resolve with shorter inserts, long-insert libraries with an average 10-kb insert size were also prepared; long-insert library generation was less efficient and required a PCR amplification step with standard methods (Asan et al. 2012). Paired-end sequencing for all libraries was performed on the Illumina HiSeq 2000 platform. Strict quality filtering of short-insert sequences yielded 37-fold sequence coverage on average.

Paired-end reads from (non-PCR-amplified) short-insert libraries were analyzed with a software pipeline designed for efficient identification of recombination breakpoints and any other sequence variation relative to the parental reference (Supplemental Fig. S1). Reads with *loxPsym* sequence were analyzed separately for direct evidence of recombinations and classified as “parental junctions,” “novel junctions” involving a recombination between pairs of designed *loxPsym* sites, or “off-target junctions” involving the recombination of a designed *loxPsym* site with any off-target sequence. Recombinations not involving *loxPsym* sites were termed “ectopic rearrangements.”

Novel junctions and copy number variation were then used to classify underlying events as deletions, inversions, insertions, tandem and nontandem duplications, and more complex rearrangements.

### Absence of off-target and ectopic recombinations

We first established that the sequenced nonsynthetic chromosomes of the JS94 parental strain matched the BY4741_v2 reference sequence (see Methods). We then assessed whether Cre generated semi-ectopic recombinations between native *loxPsym* sites and cryptic *loxP* sequences in the yeast genome, which has been reported to occur, albeit at an extremely low frequency (Sauer 1992). We found no evidence for such events, possibly because exposure to Cre was limited or because Cre sites engineered for SCRaMbLE were far more accessible than cryptic sites. Across the 64 SCRaMbLE strains, all 612 distinct novel junctions involved designed *loxPsym* sites. The average read depth of 21.5 for these novel junctions was not appreciably different from the average of 21.2 for the 2384 sequenced parental junctions. In contrast, the maximum read depth for any off-target *loxPsym* recombination was only two, suggesting these reads represent ligation artifacts rather than real in vivo recombination events.

We then examined evidence for ectopic recombinations not involving *loxPsym* sites. The average read depth for nonsynthetic nuclear chromosomes was 36.1, greater than the read depth for *synIXR* and possibly reflecting greater recovery of linear versus circular chromosomes. Previous studies using comparative genome hybridization and quantitative PCR also found reduced recovery of *synIXR* (Dymond 2011). The maximum read depth for any putative ectopic recombination was essentially the same for the parental strain (maximum read depth, eight) and the SCRaMbLE strains (maximum 10 across all 64 SCRaMbLE strains), and far less than the average read depth. Furthermore, 80.8% of the reads supporting putative ectopic recombinations involved purely mitochondrial genome sequence, which, due to mitochondrial copy number, has a much higher average read depth of 562. Alternative explanations for these reads include mitochondrial heteroplasmy and sequencing errors associated with the highly AT-rich mtDNA. In summary, we find no strong evidence for off-target or ectopic recombinations caused by SCRaMbLE.

### Full sequence reconstruction

**Figure 2.**
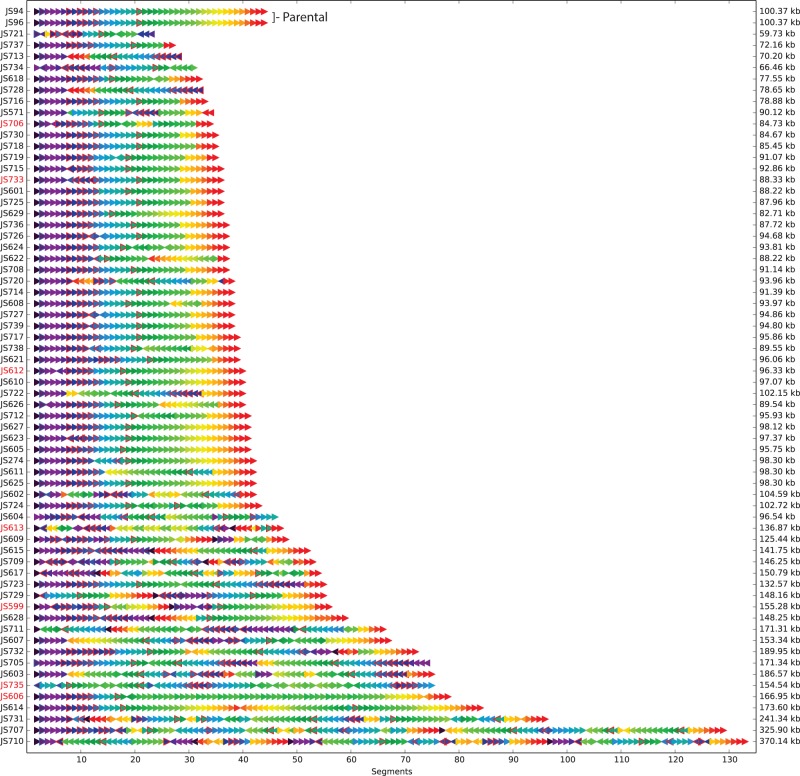
Rearrangements were observed in *synIXR* SCRaMbLE strains. Each SCRaMbLE strain is represented as a sequence of arrows. The color of each arrow indicates the segment number in the parental chromosome, and the direction of the arrow represents the orientation (SCRaMbLEgram visualization). A red border denotes a segment containing an essential gene. The names of slow growth strains are indicated in red text.

The *synIXR* SCRaMbLE chromosomes show dramatic structural diversity, depicted as “SCRaMbLEgrams” depicting the recombination endpoints observed in at least one SCRaMbLE strain (Supplemental Fig. S4) and the segment order and orientation for each strain (Fig. 2). Each strain had a unique structure; the diverse recombinations and resulting genomes support the use of SCRaMbLE to generate combinatorial diversity through random recombinations between *loxPsym* sites. Simple deletions ranged in length from one to 16 segments, or 135 to 41,999 bp. The largest simple inversion was in JS611, involving 20 segments and extending over 38,925 bp. The strain JS613, with the greatest number of simple recombinations that could be unambiguously mapped, had eight simple deletions and two simple inversions. Compared with uninduced *synIXR*, 6-h induction of *synIXR* led to 10-fold fewer viable colonies, 12 h induction to about 100-fold fewer colonies, and longer induction periods to 1000-fold fewer colonies (Supplemental Fig. S9C; see Dymond et al. 2011), providing indirect but strong evidence for more events with longer induction periods.

The observed *synIXR* junctions were then used as inputs to Euclidean path algorithms to reconstruct the rearranged chromosomes. These algorithms require only linear time to check the existence of a sequence consistent with the observed *loxPsym* junctions (Hopcroft and Tarjan 1971) and, then, near-linear time to reconstruct feasible solutions. For 39 of the scrambled *synIXR* strains, junctions observed in the 500-bp library determined a unique feasible reconstruction (Supplemental Table S1). The remaining strains had ambiguous structures supported by multiple feasible solutions because the insert size was insufficient to resolve rearrangements involving large duplications and higher amplifications. Strain JS735, for example, has 41 parental junctions, 33 novel junctions, 23 duplicated segments, and 1,732,332 solutions feasible based on junctions from short inserts.

We therefore used long-insert libraries to constrain the feasible solution and found this approach to be powerful. Of the 25 strains with multiple feasible solutions, 10 were chosen for long-insert sequencing. Each of these strains had at least 100 feasible solutions with the short-insert library, and two had over 100,000 solutions. Enforcing consistency with the long-insert reads reduced the number of solutions to four or fewer for each sequenced strain, except for JS707, whose over 1 million feasible solutions from short inserts were reduced to 48 feasible solutions.

**Figure 3.**
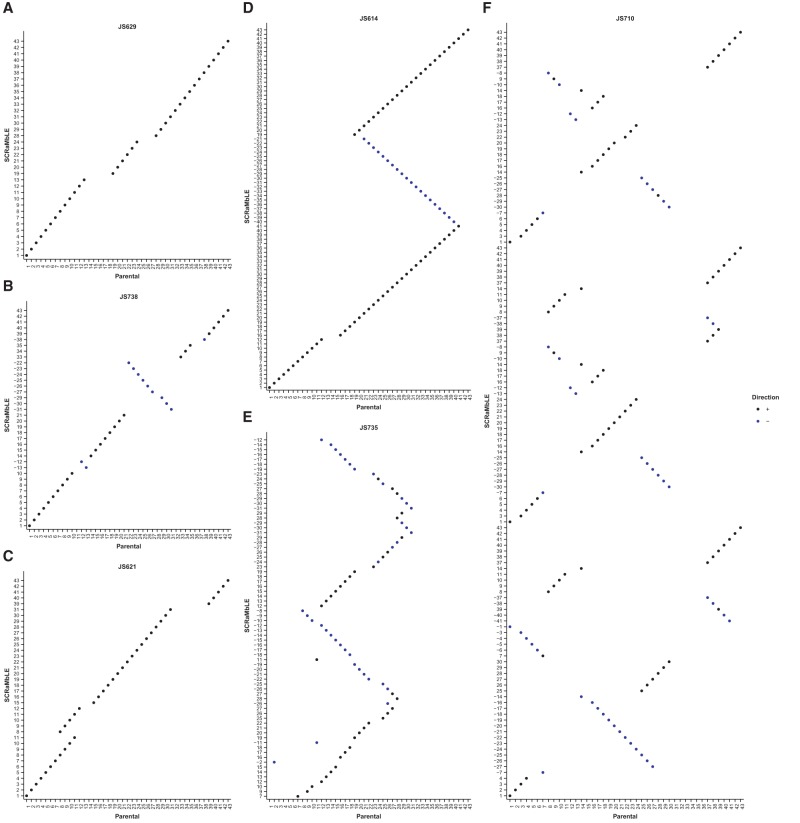
Dot-plots illustrate the pattern of the rearrangements observed for six SCRaMbLE strains. In each case, the order of segments in the SCRaMbLE strain (*y*-axis) is compared with the parental order (*x*-axis), with orientation either the same (black dot) or inverted (blue dot). (*A*) Simple deletions in JS629 create gaps. (*B*) Inversions in JS738 appear as blue stripes perpendicular to the main diagonal. (*C*) Tandem duplications in JS621 create parallel stripes. (*D*) Inverted duplications in JS614 create alternating perpendicular and parallel stripes. (*E*,*F*) Increasingly complicated patterns in JS735 and JS710 arise from recombination events within previous events, particularly when duplications are already present.

**Figure 4.**
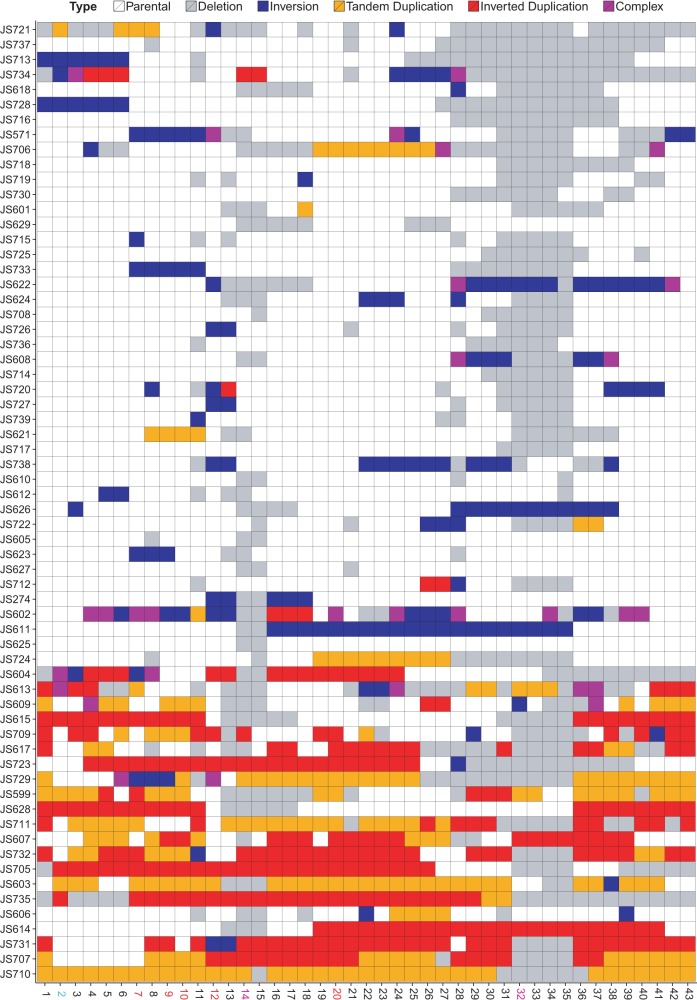
The fate of each segment in each strain is indicated as deleted (gray), inverted (blue), tandem duplication (orange), inverted duplication (red), complex (purple), or unaffected by any SCRaMbLE event and remaining at copy number 1 (white).

Different types of recombination events lead to specific patterns of self-homology, readily visualized using a representation similar to a dot-plot (Fig. 3). In this representation, the segments of the SCRaMbLE genome are plotted against the segments of the parental genome in the original order. Tandem duplications appear as multiple stripes parallel to the main diagonal; inversions are perpendicular. Recombination events were classified as deletions, inversions, tandem and inverted duplications, and more complex rearrangements with respect to the parental *synIXR* sequence (Fig. 4).

### SCRaMbLE deletions identify genes required for high fitness

**Figure 5.**
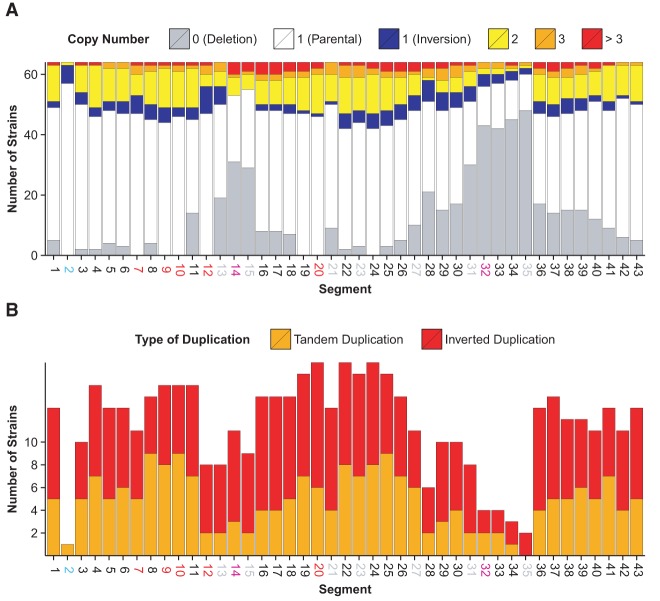
(*A*) The distribution of copy number for each segment is shown across strains. (*B*) Each segment was involved in at least one duplication event.

Most retained segments are at a single copy (Fig. 5A), and as expected, essential genes and the centromere are retained in all strains. Segments 19 and 24, which lack essential genes but contain genes required for fast growth, are also retained in all strains. The *MRS1* gene in segment 19 is essential for mitochondrial gene expression (Giaever et al. 2002). Segment 19 is also immediately upstream of essential gene *SEC11* on segment 20, and each strain also retains the 19R-20L junction; segment 19 might therefore contain regulatory elements required for proper *SEC11* expression. Segment 24 contains *YVH1*, and *YVH1* knockouts confer slow growth (Giaever et al. 2002).

Two additional genes on *synIXR* have known slow-growth phenotypes (Giaever et al. 2002): *IST3* (segment 6) and *MSL1* (segment 9). Segment 9 also contains essential gene *PRI1*. We therefore used the varying fitness of SCRaMbLE strains to determine whether segment 6 could also be identified as being required for high fitness. Each segment was tested for a higher frequency of deletion in the seven slower-growing strains (JS599, JS606, JS612, JS613, JS706, JS733, JS735) compared with the 57 high-fitness strains (Supplemental Fig. S2; Supplemental Table S2). Only deletions of segment 6 reached statistical significance (three deletions in slow-growing strains vs. present in all high-fitness strains, Fisher's exact one-sided *P*-value = 8.0 × 10^−4^ vs. 5% family-wise error threshold 1.2 × 10^−3^ for testing 43 segments).

Auxotrophy screening previously performed on the strains chosen for study generated deletion peaks close to segment 14 (containing *MET28*) and segment 32 (*LYS1*) as expected (Fig. 5A). The segment containing *MET28* occurs close to essential genes in segments 12 and 20; accordingly, deletions of *MET28* do not extend beyond these narrow confines. Deletions of *LYS1* can be much larger, with the closest essential segments in the circular chromosome being 20 and 2.

### Duplications and higher amplifications are surprisingly widespread

While the SCRaMbLE system was designed primarily to generate deletions and inversions, duplications were also found to be surprisingly frequent. At least one duplication occurred in 31 of the 64 *synIXR* SCRaMbLE strains, and each of the 43 segments is amplified in at least one strain (Fig. 5B). Prolific amplifications, sometimes spanning most of the surviving regions of *synIXR*, suggest that duplications of genes on *synIXR* do not incur a strong fitness defect, even for essential genes (*P*-value: 0.329, two-sided *t*-test for the number of strains with essential vs. nonessential segments amplified).

Amplifications may arise from the double rolling circle mechanism, which amplifies the 2-micron plasmid (Futcher 1986, 1988; Volkert and Broach 1986) and which has recently been used in engineered systems as a model for gene amplifications in cancer (Watanabe et al. 2011). Double rolling circle amplification may occur when *loxPsym* sites recombine across a replication fork within a DNA replication bubble, creating a topology in which replication forks travel in the same direction around the bubble until one is reversed by a second recombination. Unequal crossing over between two *loxP* sites during the late S or G2 phases is another mechanism that can generate duplicated regions.

Segmental copy number amplifications can be very large, with one estimated at as high as 11-fold. This amplification of the *DAL* gene cluster in strain JS606 contained an origin of replication and may represent an autonomously replicating derived plasmid in combination with *synIXR*. Consistent with this possibility, this large amplification may be unstable, as subsequent RT-PCR analysis of independent colonies from JS606 gave a range of copy number estimates (Supplemental Table S5). In JS603, the observed duplication of segment 2, containing the *synIXR* centromere, is most compatible with the presence of two distinct BACs, each containing one centromere, another possible by-product of double rolling circle amplification.

### Strains undergo repeated recombination events of varying types, consistent with equal frequencies of deletions and inversions and consistent with DNA looping

**Figure 6.**
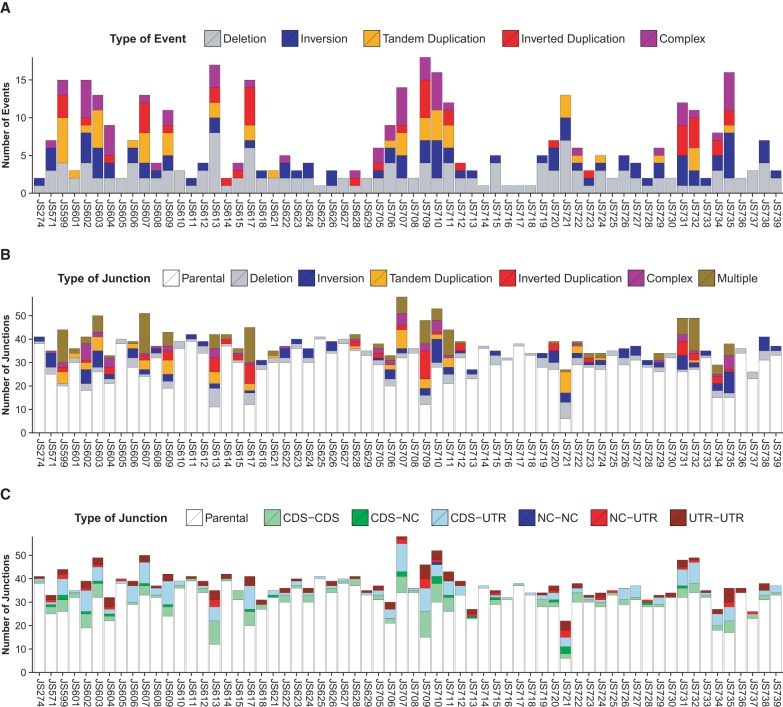
(*A*) The number of SCRaMbLE events of each type is depicted for each strain. While the number of events per strain is 6.2 ± 4.9, strains with duplications experience far more events overall. (*B*) Each event can lead to one or more novel junctions. The junctions observed in each strain are classified as parental, arising from specific types of events, or arising from multiple events (e.g., a deletion followed by an inversion) or have origins too complex to classify. (*C*) Novel junctions can create genome structures not observed in nature, including convergent coding domains lacking proper UTRs.

Phenotypic selection required each strain to undergo at least one deletion event at a selectable marker; most strains exhibit multiple additional events (Fig. 6A). Each event leads to novel junctions not observed in the parental chromosome (Fig. 6B), and novel junctions are in turn characterized by the type of DNA sequence abutting each *loxPsym* half-site: CDS, 3′ UTR, or noncoding (Fig. 6C).

**Figure 7.**
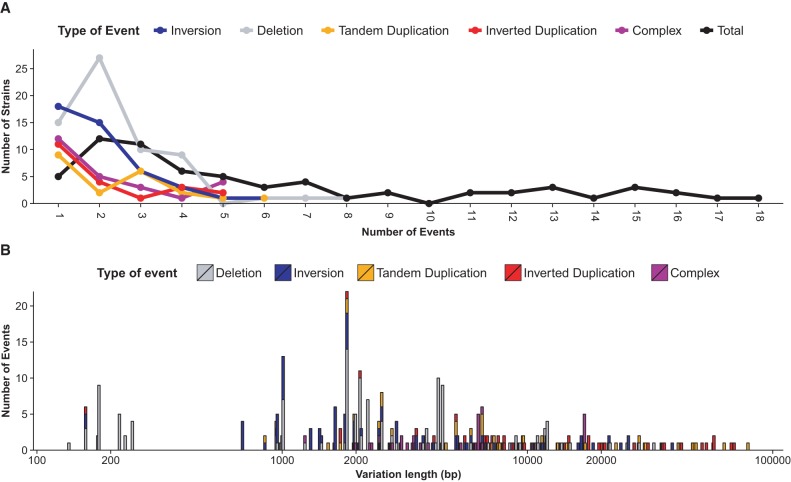
(*A*) The number of events per strain has a long tail, with strains observed having 17 and 18 distinct recombination events. (*B*) Events are more likely at short distances but continue to be observed for long separations between recombination sites. There is a preponderance of deletions over inversions at the short lengths.

While the mean number of events per strain was 6.2 ± 4.9 (Fig. 6A), the distribution of events per strain has a long tail, with 18 events observed in one strain (Fig. 7A). The *loxPsym* site was designed to permit recombination in either orientation, which, neglecting fitness effects and subtle differences in DNA bending required for deletion versus inversion, should give equal numbers of these types of events. While 156 total deletions were observed, 63 of these were required by phenotypic selection. The remaining count of 93 deletion events is indistinguishable from the 89 total inversion events (*P* = 0.83, Poisson test for rates). Similarly, 50 tandem duplications and 44 inverted duplications were observed, consistent with roughly equal frequencies (*P* = 0.66, Poisson test for rates).

Recombination event frequencies decay with distance, calculated as the distance between the *loxPsym* sites prior to an event (Fig. 7B). Deletions are further limited in length by the requirement to retain essential genes. Inversions also decay with length, consistent with statistical probabilities of DNA looping (Hagerman 1988; Rippe 2001), and more slowly than the length decay of deletions.

### Many novel junctions are tolerated with negligible fitness defects

Recombinations do not affect junctions internal to the recombination sites, and consequently, most junctions have parental origin (Fig. 6B; Supplemental Table S1). A simple deletion creates one novel junction, and a simple inversion creates two novel junctions. Thus, while the numbers of deletions and inversions are comparable, more novel junctions arise from inversions than from deletions (Fig. 6B; Supplemental Table S1). Some junctions involve *loxPsym* sites that have participated in multiple events, for example, an inversion followed by a deletion at one end. These are termed “multiple” and are particularly pronounced in strains with multiple duplications (Fig. 6B).

Each side of a junction was functionally annotated as immediately adjacent to coding domain sequence (CDS), 3′ UTR sequence (UTR), or other noncoding sequence (NC). Annotations are based on the original design of the 43 parental *loxPsym* junctions in *synIXR*, which were introduced according to prespecified rules. Each nonessential gene had a *loxPsym* site inserted exactly 3 nt after the stop codon (40 sites), including two sites flanking the centromere, creating 40 parental CDS-UTR junctions. The three remaining parental junctions are NC-NC, arising from systematic *loxPsym* replacements of LTR sequences (one site), tRNA genes (one site), and the subtelomere (one site). Novel junctions may pair a CDS with a noncognate UTR, may create converging CDS regions without terminal UTRs, or may create other genome structures with possible effects on fitness (Fig. 6C).

**Figure 8.**
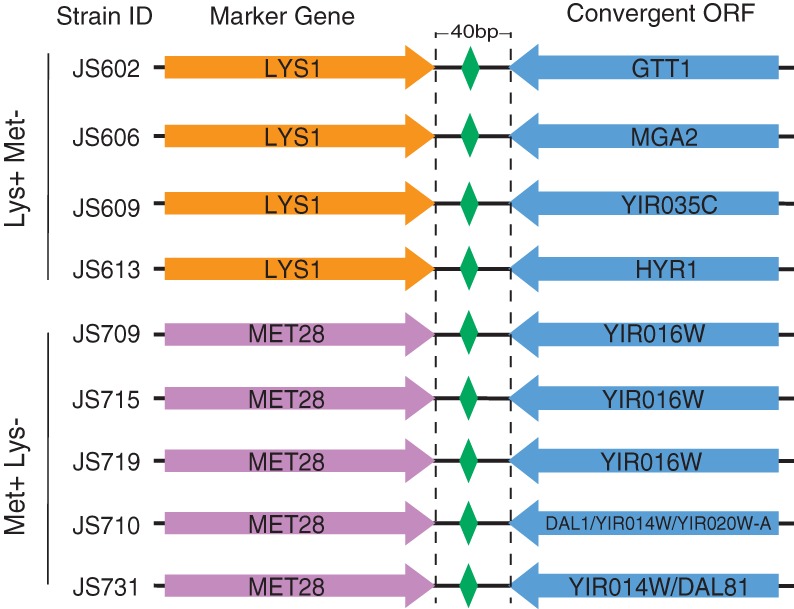
In several strains, recombinations involving auxotropic markers have generated convergeons (convergent CDS regions joined by a *loxPsym* site) that nevertheless support the prototrophic phenotype.

We examined whether novel junctions were enriched for CDS-UTR pairs, rather than CDS-CDS or CDS-NC junctions that could lead to malformed transcripts. The CDS-CDS junctions were considered likely to generate aberrant gene expression: These consist of an ORF followed by a stop codon, 6 bp of 3′ UTR sequence bisected by a single *loxPsym* site, and then an inverted ORF (Fig. 8). This bizarre arrangement of genes is not seen in nature and would presumably generate mRNAs with extremely long 3′ UTRs. Because *loxPsym* sites were introduced only after nonessential genes, however, any fitness defects may have been minor, and CDS-NC and CDS-CDS junctions were frequently observed in SCRaMbLE chromosomes. Deleterious effects of unnatural junctions were tested by comparing the observed number of novel junctions of each type (CDS-UTR, CDS-CDS, CDS-NC, UTR-UTR, UTR-NC, NC-NC) with the number predicted by a null model with the same frequency of CDS, UTR, and NC half-sites. The CDS-UTR novel junctions were underrepresented and CDS-CDS overrepresented, although these differences did not reach statistical significance (Supplemental Table S3).

We further investigated the effect of unnatural junctions by assessing the ability of *LYS1* and *MET28* to support Lys^+^ and Met^+^ phenotypes when a recombination separates the CDS from its native 3′ UTR (Supplemental Table S4). Of the 20 Lys^+^ SCRaMbLE strains, four lack the parental junction and instead have the *LYS1* CDS coupled to a convergent CDS (Fig. 8). Of the 34 Met^+^ SCRaMbLE strains, eight lack the parental junction. For three of these strains, *MET28* is joined to a noncognate 3′ UTR; for the remaining five, *MET28* is coupled to a convergent CDS (Fig. 8). These surprising results indicate that the rather unusual CDS-CDS junction type, which is never seen in nature, supports sufficient gene expression for prototrophic growth.

## Discussion

Detailed analysis of the genomes of 64 *synIXR* SCRaMbLE strains confirms that the synthetic system functions as designed. In the absence of Cre induction, the parental chromosome remained stable; studies of a yeast *synIII* strain also support this conclusion (Annaluru et al. 2014). It was important, however, to provide Cre-EBD on a plasmid; leaky expression from genomically integrated Cre-EBD led to ongoing recombinations in the absence of estradiol induction. For induced strains, no off-target action was observed: All novel junctions were recombinations at designed *loxPsym* sites, with no ectopic recombinations or other rearrangements in the 99.25% nonsynthetic genome. Short-insert libraries were sufficient to characterize the recombination junctions and copy number variations for every strain and provided unambiguous genome structures for 44 SCRaMbLE strains. Of the 25 strains with multiple solutions based on short read sequencing, 10 were selected for additional sequencing with long-insert libraries, yielding four or fewer feasible solutions for all but one. These results demonstrate that a combination of short-insert and long-insert sequencing can be sufficient to determine genome structure even for highly rearranged and duplicated SCRaMbLE genomes. The spectrum of *loxPsym* recombinations was consistent with retention of essential genes and selection for loss of phenotypic markers. Each strain had a unique genome, demonstrating the ability of SCRaMbLE to generate combinatorial diversity. Recombination hotspots and cold-spots in general may depend on selection pressure and merit continued study as SCRaMbLE is performed under different biological conditions. Deletion patterns from SCRaMbLE provided ample information to identify all annotated essential and slow-growth genes in *synIXR*. Novel junctions that swapped 3′ UTRs, including junctions coupling a CDS with a convergent CDS, had no observed effect on fitness. Segmental duplications provided evidence for double rolling circle amplification in *synIXR*; the ability of SCRaMbLE to generate gene amplifications may permit evolution of new functions.

The uniqueness of each of the 64 strains reflects the high combinatorial diversity explored by SCRaMbLE. The number of structures possible after a single event is the number of ways to select two junctions for recombination, *C*(43,2), where *C*(*n*,*k*) is the standard combinatorial factor *n*!/*k*!(*n–k*)!, multiplied by two for the choice of inversion versus deletion. Many deletions will lack essential genes and be lethal. Considering just inversions and assuming six SCRaMbLE events, the total number of trajectories is 903^6^ = 5 × 10^17^, setting an order of magnitude for the combinatorial space. While it is not surprising that independent samples from this space would be unique, it is important to note that the evolution was not independent and that no single clone dominated the evolving population.

### Full reconstruction

Short-insert libraries are sufficient to characterize copy number variation and novel junctions in the SCRaMbLE strains. We used PCR-free library preparation to avoid chimeric reads caused by mishybridization of *loxPsym* sequences. Read depth for the *synIXR* was ∼80% of the depth for the native chromosomes, which may reflect difficulties in recovery of DNA from circular versus linear chromosomes with standard protocols, as we previously showed by other means. The main weakness of the short-insert sequencing was its inability to yield unique reconstructions in the face of large duplications, which in general require large-insert libraries that are not PCR-free. We therefore explored a hybrid approach using large-insert libraries to constrain the reconstructions from short inserts. This approach was successful in vastly reducing the number of feasible reconstructions, in some cases from over a million to only a handful.

### Stability of parental genomes and derived strains

The sequenced strains provide strong evidence for stability of the parental genome prior to SCRaMbLE induction, as does other recently performed work (Annaluru et al. 2014), and continued stability of derived strains after SCRaMbLE is deactivated. For episomal Cre-EBD, each of the sequenced genotypes is consistent with the previously measured phenotype, indicating that no additional markers have been lost across all the strains. There is evidence, however, for leaky expression of Cre-EBD integrated genomically, with the two corresponding strains undergoing recombination in the absence of induction. These results suggest that leaky expression leads to low levels of recombinase activity over an extended selection timeframe. This may actually prove quite useful for long-term evolution experiments, where too high recombination rates can become a liability.

### Designed recombination sites and off-target activity

In the observed data, recombinase activity is entirely limited to designed *loxPsym* sites, with no evidence suggesting off-target activity or recruitment of cryptic sites in the nonsynthetic genome. While off-target activity of Cre has been documented previously, the rate for designed SCRaMbLE recombinations must be many orders of magnitude larger, making off-target recombinations rare to nonexistent in practice in this context. Similarly, ectopic rearrangements or breakpoints are not observed at frequencies greater than for parental strains lacking recombinase.

### Recombinations and fitness

Retention and deletion of genome segments is strongly driven by imposed selections and known essential genes. Significantly, all genes known to be required for fast growth were readily identified by statistical tests of SCRaMbLE deletions. Additional fitness constraints were difficult to identify. In particular, recombination events that separated a CDS from its downstream UTR, or even that paired a CDS with a second CDS rather than a UTR, showed no evidence for fitness penalties.

In principle, analysis of additional strains should provide information about synthetic lethal fitness defects. Suppose that a synthetic region includes *T* total nonessential genes, with *P* = *T*(*T* − 1)/2 total pairs to test for synthetic lethal interactions, and that *N* independent SCRaMbLE strains are available for analysis, of which *D* have both genes deleted through SCRaMbLE events and *N*–*D* have at least one gene present. Using *d*_1_ to represent the fraction of strains that have lost the first gene and *d*_2_ as the fraction having lost the second gene, the probability under the null for losing both genes is the product *d*_1_*d*_2_, denoted *d*^2^. The observation *D* = 0 provides evidence for a synthetic lethal interaction. Under the null hypothesis, the probability of *D* = 0 is (1 − *d*^2^)^*N*^, which when *d*^2^ is small is approximated well by exp(−*Nd*^2^). Significance at a *P*-value of 0.05 after testing *P* total pairs requires exp(−*Nd*^2^) < 0.05/*P*, or *N* > [2 ln *T* + ln 10 ]/*d*^2^. For the *synIXR* strains, *T* = 43 regions, *d* is ∼20% (biased upward by selection for auxotrophy), and *N* ≈ 250 strains would be required. The number of strains required grows slowly as the logarithm of the size of the synthetic region. For a typical chromosome with *T* ≈ 300 nonessential genes, and assuming a smaller fraction *d* ≈ 5% gene loss in the absence of auxotrophic selection, approximately 6000 strains would be required. For an entire synthetic genome with *T* ≈ 5000 nonessential genes and *d* ≈ 5% gene loss, approximately 8000 strains would be required. Experiments of this scale could potentially be accomplished using new single-cell sequencing methods (Klein et al. 2015; Macosko et al. 2015; Rotem et al. 2015) to interrogate a population of SCRaMbLE strains.

While naturally occurring *loxP* sites are asymmetric and can generate only deletions or inversions, symmetrical synthetic *loxPsym* sites give an equal mix of deletion and inversion products in vitro (Hoess et al. 1986). Our ratio of deletions to inversions in the *synIXR* SCRaMbLE strains is entirely consistent with equal likelihood of deletion and inversion.

Duplications of sequence and higher-order amplifications are observed in many strains and do not appear to cause substantial fitness defects. While short duplications may arise from recombinations during S or G2 phase when multiple chromosome copies are present, long duplications almost certainly arise through the double rolling circle mechanism. Duplications in SCRaMbLE chromosomes are particularly welcome because duplicated regions are much more likely than deletions to give rise to a gain of function, and thus SCRaMbLE is likely to give rise to a broader range of phenotype diversity than simple loss of function.

### Conclusions

SCRaMbLE combines designed recombination sites with controlled expression of recombinase for efficient, robust generation of genomic diversity. Deep sequencing of yeast strains before and after SCRaMbLE demonstrates that the parental genome is stable, with no evidence for recombinations outside the designed sites. Observed variations include deletions, inversions, duplications, and UTR-swaps, as well as odd structures not observed in the native genome, such as two convergent CDSs separated by only a *loxPsym* site. Deletion frequencies readily identify genes required for high fitness. Variations in gene structure, particularly those that disrupt junctions between CDS and UTR regions, are frequently observed and do not appear to cause fitness defects. The double rolling circle mechanism likely contributes to the amplification of large regions, which is predicted to produce useful “gain-of-function” phenotypes. This system will be valuable in combinatorial exploration of genomic diversity for gene–gene and gene–environment interactions and phenotype-based selection.

## Methods

### *synIXR* SCRaMbLE strain generation and Met/Lys auxotrophy selection

Seven independent colonies encoding *synIXR*-BAC were used as the sole source of this chromosome arm (Supplemental Table S1). All but one of the SCRaMbLE strains were generated by transforming with an episomal, *URA3*-tagged Cre-EBD; the single exception (JS274) had integrated Cre-EBD genomically at the *HO* locus. In each case, SCRaMbLE was induced for 4 h in synthetic medium lacking uracil in the presence of 1 µM estradiol. Single colonies were then isolated by streaking SCRaMbLE cultures onto rich yeast medium (YPD). To ensure loss of the Cre-EBD construct, patching on YPD was followed by a second round of single colony purification performed on YPD medium. Colonies were then tested for resistance to FOA. Finally, *met28* and/or *lys1* deletion mutants were identified by replica plating onto synthetic medium lacking methionine or lysine (Dymond et al. 2011). All chosen isolates were additionally subjected to serial spot dilution assays on YPD and synthetic complete medium lacking methionine or lysine to verify the genotype.

### Library preparation

The sequencing libraries were made without PCR amplification to avoid creating junction artifacts through crossover PCR driven by hybridization of *loxPsym* sites. In addition, in order to accurately estimate copy number of target regions, amplification-free sequencing decreased the likelihood that an appreciable proportion of these sequences would be duplicates and preserved a more even distribution of read coverage across the targeted sequencing regions (Korbel et al. 2007). Cultures of reference and SCRaMbLE strains were grown in 30 mL YPD at 30°C until saturated. DNA was extracted by the glass beads method (Xiao 2006), and the amplicon-free libraries were prepared following the Illumina protocol. Ten micrograms of DNA was sheared using a Covaris S2 to an average length of 500 bp, end-repaired, and ligated to Illumina paired-end adapters. Ligated fragments were selected on agarose gels and purified to yield the corresponding libraries. A large amplicon library of average length 10 kb was also prepared for 10 strains by published methods (Asan et al. 2012). All constructed libraries were sequenced on the Illumina HiSeq 2000 platform.

### Mapping to the parental genome

Illumina HiSeq 2000 paired-end short reads were trimmed to remove adapter sequence. We then used strict filtering to remove <100 bp or duplicated reads. Reads with unknown bases or with bases having a Phred-score below seven were removed. Filtered reads were mapped to the *synIXR* reference sequences JN020955 and using the Soap short-read aligner (http://soap.genomics.org.cn/) for standard data processing of reads mapping to the parental genome with no rearrangements (Li et al. 2009). For the nonsynthetic chromosomes, BY4741 served as the reference (http://downloads.yeastgenome.org/sequence/strains/BY4741/BY4741_Toronto_2012/). Paired-end reads that could not be mapped directly were analyzed using Bowtie (Langmead et al. 2009) using standard settings.

### Splitting reads for junction identification

Reads containing *loxPsym* sites that did not map to the parental genome, with at least 15 bp of sequence flanking the *loxPsym* site, were then trisected into a *loxPsym* site and two single ends, which remain associated with the paired sequence from the other end of the fragment. The two single ends were then aligned to the reference using Bowtie 2 (Langmead and Salzberg 2012) by single-end mapping to determine a novel *loxPsym* junction, with at least three reads required for support.

### Ectopic and parental structural variant detection

Reads without *loxPsym* sites that did not map to the parental genome were directly split into two ends. In order to precisely locate a possible breakpoint, the split site was scanned over all intermediate positions at least 15 bp from the ends of the read. Then the two ends were aligned to the reference using Bowtie 2 by single-end mapping with parameter –k 100 for breakpoint detection, which could provide direct evidence of an ectopic recombination. For each identified breakpoint, a 5-bp error range was allowed. For a pre-existing parental variation relative to the BY4741_v2 reference, a 15-bp error range was allowed and at least two reads per sample were required for support to identify pre-existing breakpoints.

To detect recombination events outside of the designed target *loxPsym* site, we also applied CREST (Clipping REveals STructure), an algorithm that detects genomic structural variations at base-pair resolution (Wang et al. 2011). A sequence read that spans a breakpoint will have partial alignment to both sides of the junction. Therefore, BWA (Li and Durbin 2009) was used first to perform local alignment to all generated reads after quality control. The unaligned portion of reads were masked by a process termed “soft-clipping” because the unaligned subsequence is retained but not trimmed even though it does not map to the current genomic location. Then soft-clipped reads were extracted from the binary alignment/map (BAM) file. First, soft-clipped reads at a putative breakpoint were assembled into a contig; the contig was then mapped against the reference genome to identify candidate partner breakpoints; then all possible soft-clipped reads were identified and assembled into a contig, followed by an alignment of the contig derived from the partner back to the reference genome. Finally, a match to the initial breakpoint was performed to check whether the breakpoint prediction was correct. All breakpoints with read depth close to the average depth involved recombinations between pairs of designed *loxPsym* sites.

### Copy number variation

Copy numbers were estimated to detect deletions, duplications, and higher amplifications. The average sequencing depth of *synIXR* was 29.02. We then marked as deleted every segment with read depth of less than five. After proceeding with the copy number estimation algorithm below, the average read depth for deleted segments was 0.27 and the maximum was 4.98. Segments 1 and 38 exhibit a read depth close to five when deleted because reads from homologous regions in the genome are erroneously mapped.

To account for possible systematic bias in sequencing depth due to confounding factors from library preparation and mapping, we used an iterative algorithm to refine the copy number estimation and the potential bias. The sequence comprised segments numbered 1 through *L* = 43. The observed read depth of segment *i* in strain *t* was denoted *D*_*ti*_ and in the reference strain *r* with no rearrangements was *D*_*ri*_. The inferred copy number for this segment in strain *t* was denoted *N*_*ti*_ and was *N*_*ri*_ = 1 for the reference strain. Successively, for each segment *i*, we calculated the ratio

$$N_{ti}/N_{ri}=k(D_{ti}/D_{ri}).$$

The constant *k* reflects the overall ratio of read depth. It was determined self-consistently from a different segment *j*, *j* ≠ *i*, as

$$k=(N_{tj}/N_{rj})(D_{rj}/D_{tj}).$$

Using segment *j*, the copy number for segment *i* of sample *t* was estimated as

$$N_{ti}/N_{ri}=(N_{tj}/N_{rj})(D_{rj}/D_{tj})(D_{ti}/D_{ri}).$$

Since the reference copy number is known to be one for each segment,

$$N_{ti}=(N_{tj}D_{rj}/D_{tj})(D_{ti}/D_{ri}).$$

Iterations were initialized with *N*_*ti*_ = 1 and successively computed an estimation matrix *M* with element *M*_*ij*_ = *N*_*ti*_ using segment *j* as the reference. The value of *N*_*ti*_ was updated to be the maximum of the estimates, max*_j_ M*_ij_.

To evaluate the confidence of the estimate, we defined the coefficient of variation for each segment *i* as

$$CV_{i}=\mathrm{stdev}(EM_{i})/\mathrm{mean}(EM_{i}).$$

At each iteration, the copy number estimation was updated by selecting the most frequent value for *EM*_*i*_, and we iterated the process until each *CV*_*i*_ converged with a tolerance ε < 0.25.

### Structural variation and genome reconstruction

Each rearranged genome, denoted *T*, was represented by an undirected graph *G* = (*V*,*E*). The vertices *V* represented the segments of *T*, integer values with magnitude one through the total number of segments *L* (in this case 43) and sign indicating direction relative to the reference. The edges *E* represented the junctional-reads for adjacent segments. For each tandem duplication of segment *i* with copy number *N*_*i*_, *N*_*i*_ − 1 vertices were added to the graph and connected as a ring.

Given this graph *G*, the rearranged genome sequence was reconstructed by finding a cycle that visits each edge in *G* once, termed a Eulerian cycle over *G* (Garey and Johnson 1979). Algorithms based on Eulerian cycles have become standard in DNA sequence assembly, with many efficient methods (Schatz et al. 2010). We first checked for the existence of a Eulerian cycle by verifying that each vertex has an even degree and all belong to one and only one connected component; these operations can be performed in linear time (Hopcroft and Tarjan 1971). While it is possible that a mixture of two distinct genomes may lead to no cycles being found, in each case we found at least one Eulerian cycle.

Multiple Eulerian cycle solutions were found in some cases. These result from rearrangements that extend farther than the length of the paired-end library and introduce ambiguities similar to genome assembly with long repeats. The multiple solutions have equal length and identical composition of segments and segment junctions.

### Long-insert libraries

Paired-end reads from long-insert libraries with fragment sizes of ∼10 kb were used to constrain the solutions for the 10 strains with the most complex rearrangements. Quality thresholds for sequencing reads from the 10-kb library were the same as for the 500-bp library. The reads of ∼90 bp were aligned to the reference (Ref Strain: BY4741, the original right arm sequence of Chromosome 9 was replaced with the synthetic sequence) using SOAPaligner 2.21 (Li et al. 2009), available at http://soap.genomics.org.cn/soapaligner.html, with parameters -m 7500 -x 12500 -r 1 -v 4 –R.

Theoretically, read mapping to a most likely reconstruction will lead to a very high regular paired-end mapping rate, PE/[PE + SE], where PE are paired-ends mapping with correct distance and orientation, and SE are paired-ends that map individually but with incorrect distance or orientation, without any region that has no read covering or any irregular mapping read clusters. Therefore, the regular mapping rate of reconstruction sequence is calculated for all samples’ reconstructions, and the absence of irregular mapping clusters is also checked. Reconstructions that meet both criteria are considered correct reconstructions.

Following this analysis, we reduced the number of feasible reconstructions for seven complex strains from 193,642 to 18 (strains JS613, JS711, JS732, JS607, JS603, JS617, JS731). When the solution is unique, the single reconstruction is reported. When multiple solutions are possible, the solution that is lowest in numerical sort order is reported.

### Novel junction frequencies

Novel junctions in *synIXR* SCRaMbLE strains were classified as CDS-CDS, CDS-UTR, CDS-NC, UTR-UTR, UTR-NC, or NC-NC, with each of *T* total novel junction counted only once per strain regardless of copy number. The number of CDS half-sites, *n*_*C*_, was calculated as (2× number of CDS-CDS novel junctions) + (number of CDS-UTR) + (number of CDS-NC), and similarly for the number *n*_*U*_ of UTR and *n*_*N*_ of NC half-sites. CDS half-site probabilities in novel junctions were calculated as *p*_*C*_ = *n*_*C*_/(*n*_*C*_+ *n*_*U*_+ *n*_*N*_), and similarly for *p*_*U*_ and *p*_*N*_. The expected number of CDS-CDS novel junctions under the null was then modeled as Poisson distributed with mean *T p*_*C*_^2^; the expected number of CDS-UTR novel junctions was modeled as Poisson with mean *T p_C_ p*_U_ and similarly for all six possible junction types. For each junction type, two-sided *P*-values were calculated as twice the probability of the smaller of the lower and upper tail for the observed value.

### Classifying recombination events

Reconstruction events and junctions were classified as follows. Duplication events were identified as repeating regions of the reconstruction genome. Repeating regions in the same direction were further classified as tandem duplications, and repeat regions in alternating head-to-tail orientation were classified as inverted duplications. Subregions of the nondominant direction within repeat and nonrepeat regions were identified as inversion events. Deletion events were identified as continuous regions of the wild-type genome with segment copy number of zero.

Next, each junction was considered in turn. A junction between two repeat regions or between repeat and nonrepeat regions was classified as a duplication junction. A junction between inversion and noninversion regions was an inversion junction. A deletion junction was a junction between two segments that are adjacent to the left end and the right end of a deleted region in the parental genome. Nonparental junctions not associated with the endpoints of deletion, duplication, or inversion events were classified as complex junctions. Contiguous regions of complex junctions were then defined as a single complex event.

### Semi-*synVIL* SCRaMbLE strains

CRE-EBD was integrated at the *HO* locus of semi-*synVIL*, encoding ∼30 kb of designer synthetic DNA on the left arm of Chromosome *VI* to generate strain yJS704. Strain yJS704 was streaked onto rich medium (YPD) supplemented with estradiol (1 µM) and grown for 2 d at 30°C. Four independent colonies were selected and then processed in parallel, restreaking an additional five times onto fresh estradiol-containing YPD plates. In each case, after 2 d a single independent clone was selected for restreaking of that lineage. This process was repeated in parallel five more times for a total of 12 d of estradiol exposure. The absence of the entire synthetic segment was confirmed by PCR. Semi-*synVIL* strains were also subjected to SCRaMbLE, with five strains recovered and all resulting in deletion of the synthetic region. The strain JS704 had the Cre-EBD integrated at the *HO* locus and, while not induced, lost segment 2, presumably through leaky expression of the recombinase, and is counted among the SCRaMbLE strains. One non-SCRaMbLE parental strain was also sequenced. All semi-*synVIL* strains that underwent SCRaMbLE were identical, deleting the entirely dispensible segments lying between the terminal *loxPsym* sites (Dymond and Boeke 2012). Contributing to complete loss were extended exposure to recombinase, 12 d for the semi-*synVIL* strains versus 4 h for the *synIXR* strains, and an absence of essential genes in the entire semi-*synVIL* region. Reads were analyzed by mapping to the JN020956 reference sequence and then following the same procedure outlined for *synIXR*.

## Data access

The sequence data from this study have been submitted to the NCBI Sequence Read Archive (SRA; http://www.ncbi.nlm.nih.gov/sra/) under accession number SRA124245. The individual clones have sample accession numbers SRX470091 through SRX470176; the clone identifiers used here are provided as the sample names in SRA.

## Supplementary Material

Supplemental Material
