# Supplementary material for: SCRaMbLE generates designed combinatorial stochastic diversity in synthetic chromosomes
Source: Genome Res. 2016 Jan;26(1):36–49. doi: 10.1101/gr.193433.115 (PMC4691749; doi:10.1101/gr.193433.115)
Supplement: Supplemental Material [file supp_26_1_36__index.html]

SCRaMbLE generates designed combinatorial stochastic diversity in synthetic chromosomes — SCRaMbLE generates designed combinatorial stochastic diversity in synthetic chromosomes — SCRaMbLE generates designed combinatorial stochastic diversity in synthetic chromosomes — Supplemental Material 

# SCRaMbLE generates designed combinatorial stochastic diversity in synthetic chromosomes

## Supplemental Material

**Files in this Data Supplement:**

- Supplemental Material.docx
- TableS1.xlsx
- TableS2.xlsx
- TableS3.xlsx
- TableS4.xlsx
- TableS5.xlsx
