# Supplementary material for: SCRaMbLE generates designed combinatorial stochastic diversity in synthetic chromosomes
Source: Genome Res. 2016 Jan;26(1):36–49. doi: 10.1101/gr.193433.115 (PMC4691749; doi:10.1101/gr.193433.115)
Supplement: Supplemental Material [file supp_gr.193433.115_Supplemental_Material.docx]

Supplementary Materials

Supplementary Figure 1. SCRaMbLE reconstruction pipeline 2

Supplementary Figure 2. Fisher’s Exact Test for Enrichment of Segment Deletion in Slow Growth Strains. 3

Supplementary Figure 3. The structure of synVIL. 4

Supplementary Figure 4. Rearrangements observed in synIXR SCRaMbLE strains. 5

Table S1. List of Strains 6

Table S2. Fitness Analysis 8

Table S3. Analysis of Junction Structure 9

Table S4. Analysis of Junction Structure for MET28 and LYS1 Markers 10

Table S5. RT-PCR for JS606 Duplications 11

# Supplementary Figure 1. SCRaMbLE reconstruction pipeline

#
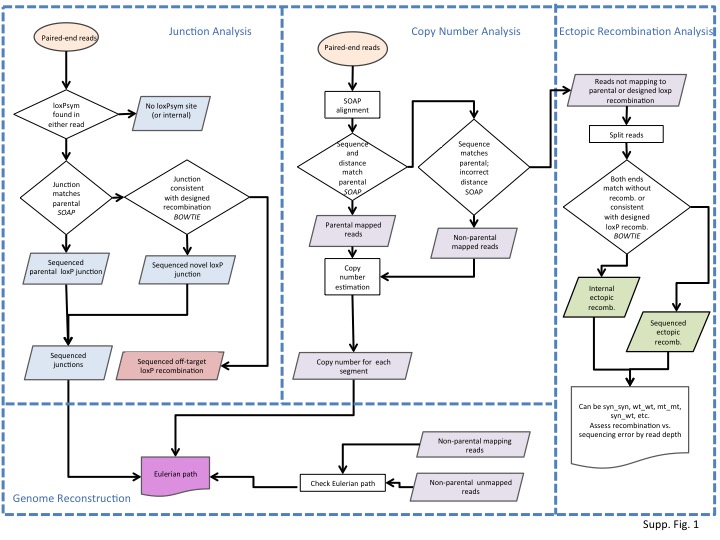


# Supplementary Figure 2. Fisher’s Exact Test for Enrichment of Segment Deletion in Slow Growth Strains.

The figure shows the p-value from Fisher’s exact test for enrichment of a specific segment in slow growth strains. Dots in red are statistically significant results at 5% FWER (threshold=1.2x10^–3^).


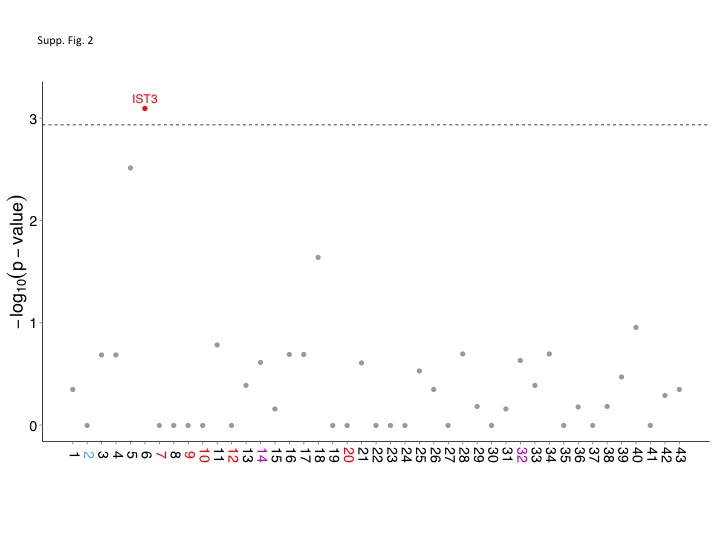


# Supplementary Figure 3. The structure of synVIL.

Bars indicate loxPSym sites (orange, numbered) and auxotrophic markers (purple). Arrows indicate essential genes (red), non-essential genes (light blue), and the chloramphenicol resistance marker (green).

# Supplementary Figure 4. Rearrangements observed in synIXR SCRaMbLE strains.

Segments that are endpoints of a recombination observed in at least one SCRaMbLE strain are connected by edges. The segment colour indicates presence of an essential gene (red), an auxotrophic marker (purple), the centromere (blue), or no essential features (white) within the segment. The edge colour indicates whether the segments were the endpoints of a deletion (grey), inversion (blue), tandem duplication (orange), inverted duplication (red), complex (purple), or are parental (black edges along the circumference).

# Table S1. List of Strains

The table reports the following information for each analyzed strain:

- Column A: strain id
- Column B: the synthetic chromosome integrated in the strain
- Column C: the phenotype
- Column D: Flag indicating if the strain has been SCRaMbLEd
- Column E: Flag indicating if CRE-EBD was integrated at the HO Locus
- Column F: Flag indicating slow growth phenotype
- Column G: SCRaMbLE Round.
- Column H: Tube for SCRaMbLE experiment.
- Column I: Strain colony number
- Column J: Total number of reads
- Column K: Total number of reads after quality control
- Column L: Total number of reads mapping to wild-type chromosomes
- Column M: Total number of reads mapping to the synthetic chromosome after bisection/trisection
- Column N: Total number of reads mapping to the synthetic chromosome after bisection/trisection
- Column O: Total number of unclassified reads.
- Column P: Maximal read depth supporting a putative ectopic recombination
- Column Q: Average sequencing depth on the wild-type chromosomes
- Column R: Average sequencing depth on the synthetic chromosome
- Column S: Total number of reads containing a loxPsym site
- Column T: Number of reads mapping on non-syn chromosome
- Column U: Number of reads mapping to parent sequence
- Column V: Number of reads mapping to parent sequence and consistent with solution
- Column W: Number of reads not mapping to parent sequence and consistent with solution
- Column X: Number of reads mapping to parent sequence and inconsistent with solution
- Column Y: Number of reads not mapping to parent sequence and inconsistent with solution
- Column Z: Maximal read depth supporting a putative off-target recombination
- Column AA: Total number of recombinations
- Column AB: Number of new junctions that are CDS-UTR
- Column AC: Number of new junctions that are CDS-CDS
- Column AD: Number of new junctions that are UTR-UTR
- Column AE: Number of new junctions that involve non-CDS or non-UTR sequence (when the loxP site wasn't in a 3' UTR)
- Column AF: Number of segments observed at copy number 1
- Column AG: Number of segments observed at copy number 2 or more
- Column AH: Number of segments deleted
- Column AI: Number of reconstructions
- Column AJ: Number of 10kb reads
- Column AK: Number of reads mapping to syn chromosome parent sequence
- Column AL: Number of reads mapping to syn chromosome with recombination, consistent with solution (loxp)
- Column AM: Number of reads mapping to syn chromosome but inconsistent with solution(loxp)
- Column AN: Final number of possible unique reconstruction
- Column AO: Solutions
- Column AP: Number of deletion events
- Column AQ: Number of deletion junctions
- Column AR: Number of inversion events
- Column AS: Number of inversion junctions
- Column AT: Number of tandem duplication events
- Column AU: Number of tandem duplication junctions
- Column AV: Number of inverted duplication events
- Column AW: Number of inverted duplication junctions
- Column AX: Number of complex events
- Column AY: Number of complex junctions

# Table S2. Fitness Analysis

The table reports counts and p-values from Fisher’s exact test for testing enrichment of deletions in slow growth strains.

The table reports the following information:

- Column A: Segment id
- Column B: Number of slow growth strains where the segment has been deleted
- Column C: Number of slow growth strains where the segment is present
- Column D: Number of normal growth strains where the segment has been deleted
- Column E: Number of normal growth strains where the segment is present
- Column F: Two tailed p-value from Fisher’s exact test
- Column G: Lower tail p-value from Fisher’s exact test
- Column H: Upper tail p-value from Fisher’s exact test

# Table S3. Analysis of Junction Structure

The table reports p-values, from a Poisson test, for enrichment or depletion of junction structures respect the expected null distribution.

# Table S4. Analysis of Junction Structure for MET28 and LYS1 Markers

The table reports the number of different junction structures involving MET28 and LYS1 markers.

# Table S5. RT-PCR for JS606 Duplications

The predicted structure of JS606 indicates that the segment region 24-26 is duplicated ~10 times. For the QPCR validation, segment 26 and segment 22 (as internal reference) were measured in both JS606 & JS94. Results are provided for several different single colonies.
